# The Other Site of Rhabdomyosarcoma

**DOI:** 10.1002/cam4.70348

**Published:** 2024-10-28

**Authors:** Leonie Kern, Anton Henssen, Angelika Eggert, Monika Scheer

**Affiliations:** ^1^ Department of Pediatric Hematology and Oncology Charité–Universitätsmedizin Berlin, Corporate Member of Freie Universität Berlin and Humboldt‐Universität zu Berlin Berlin Germany

**Keywords:** adult sarcoma, age, age‐spanning, lymph nodes, other site, pediatric sarcoma, rhabdomyosarcoma, soft tissue sarcoma

## Abstract

**Background:**

Rhabdomyosarcoma (RMS) is a rare malignant soft tissue sarcoma (STS), accounting for almost 50% of pediatric STSs. Due to its heterogeneity, RMS presents challenges in diagnosis and treatment, with prognosis varying depending on multiple factors. Tumors localized in the other site (OTH)—including the paraspinal, perianal, thoracic, abdominal, pelvic, and perineal regions—are generally classified as unfavorable. This study assesses the clinical features and prognoses of RMS in OTH locations depending on its site of origin.

**Methods:**

An explorative analysis of RMS cases from the SEER 17 database 2000–2020 was conducted. Patients of all ages with histologically confirmed RMS as primary malignant disease classified under OTH, were included. OTH was categorized in four granular site classifications. Overall survival (OS) and disease‐specific survival (DSS) were analyzed using Kaplan–Meier estimators. Factors independently influencing survival, including a site classification model presented in this study, were identified through Cox regression analysis.

**Results:**

Out of 4168 patients with RMS, 990 cases of RMS with the OTH site met the inclusion criteria. The median age was 16 years. The predominant histological subtypes were embryonal (33.0%) and alveolar (25.5%). Most tumors were ≥ 5 cm (median 9 cm) and located primarily in the pelvic region (41.5%). The 3‐, 5‐, and 10‐year OS rates were 45.4% ± 3.332 (95% CI), 40.7 ± 3.332, and 38.6% ± 3.332, respectively, while DSS rates were 43.3% ± 3.136 (95% CI), 38.3% ± 3.136, and 35.1% ± 3.332. In the multivariate analysis age, histological type, site in a granular categorization, stage, regional lymph node examination, and regional lymph node involvement (pathologically proven) were independently associated with survival. Through both univariate and multivariate analyses, an OTH favorable group could be established. The OTH favorable group consists of the anal region, gallbladder and biliary tract, and breast.

**Conclusion:**

RMS in OTH shows significant differences in prognosis, putting the current categorization as unfavorable into question and making a more detailed classification necessary. Furthermore, pathological regional lymph node assessment is specifically in the OTH localization recommended.

## 1 Introduction

Rhabdomyosarcoma (RMS) is a malignant soft tissue sarcoma (STS) that, despite its overall rarity, constitutes for nearly 50% of all pediatric cases of STS. While RMS can manifest at any age, its prominence in adult oncology remains limited, primarily due to its relatively low incidence rates [1, 2, 3, 4]. RMS remains a complex and heterogenous malignancy, with various histological subtypes, primary sites, and clinical and molecular characteristics. The heterogeneity of RMS poses significant challenges in terms of diagnosis, as well as treatment, and leads to a variability in clinical outcomes despite advances in treatment [5, 6, 7]. However, the site of the primary tumor has commonly been recognized as a crucial prognostic factor, leading to the classification of different sites as favorable or unfavorable. The site classification “other” (OTH), which includes tumors arising from paraspinal or perianal regions, as well as those originating from the thorax, abdomen, pelvis, or perineum, is considered unfavorable [8, 9, 10, 11, 12, 13, 14]. Given the diversity of this site, questions have been raised regarding the necessity for a more specific subdivision of OTH. Understanding how primary tumor site of RMS affects disease prognosis is important for improving treatment strategies and refining risk assessment methods. As OTH accounts for at least 13.8% of all RMS [15], a closer look at this localization is important. In the present study, an analysis of RMS cases was performed, using the publicly accessible US‐American Surveillance, Epidemiology and End Results (SEER) database, across all ages, in order to characterize clinical features and outcomes of different sites within the site classification OTH. The aim is to develop classification systems for the various localizations within this diverse group and to assess their overall definition as unfavorable.

## 2 Material And Methods

Study data was acquired through the SEER 17 registries. The database includes patient and clinical data, as well as information on survival, recorded in the United States from the years 2000 until 2020. The SEER database is categorized by histologic type and not, as with many other cancer registries and groupings, by the site of the cancer.

Included were tumors with malignant behavior and patients with known age. Furthermore, tumors classified as IX. Soft Tissue And Other Extraosseous Sarcomas based on the International Classification of Diseases for Oncology, version 3 (ICD‐O‐3) morphology: RMS were selected. This included 8900 RMS, NOS, 8901 Pleomorphic RMS, adult type, 8902 Mixed type RMS, 8910 Embryonal RMS, NOS, 8912 Spindle cell RMS, 8920 Alveolar RMS and 8921 RMS with ganglionic differentiation. Cases reported with death certificate only/autopsy only were excluded.

All available information on primary site of disease according to the variable “Primary Site–labeled” was reviewed and classified according to the international RMS site classification system [8, 9, 10, 13, 14].

**FIGURE 1:**
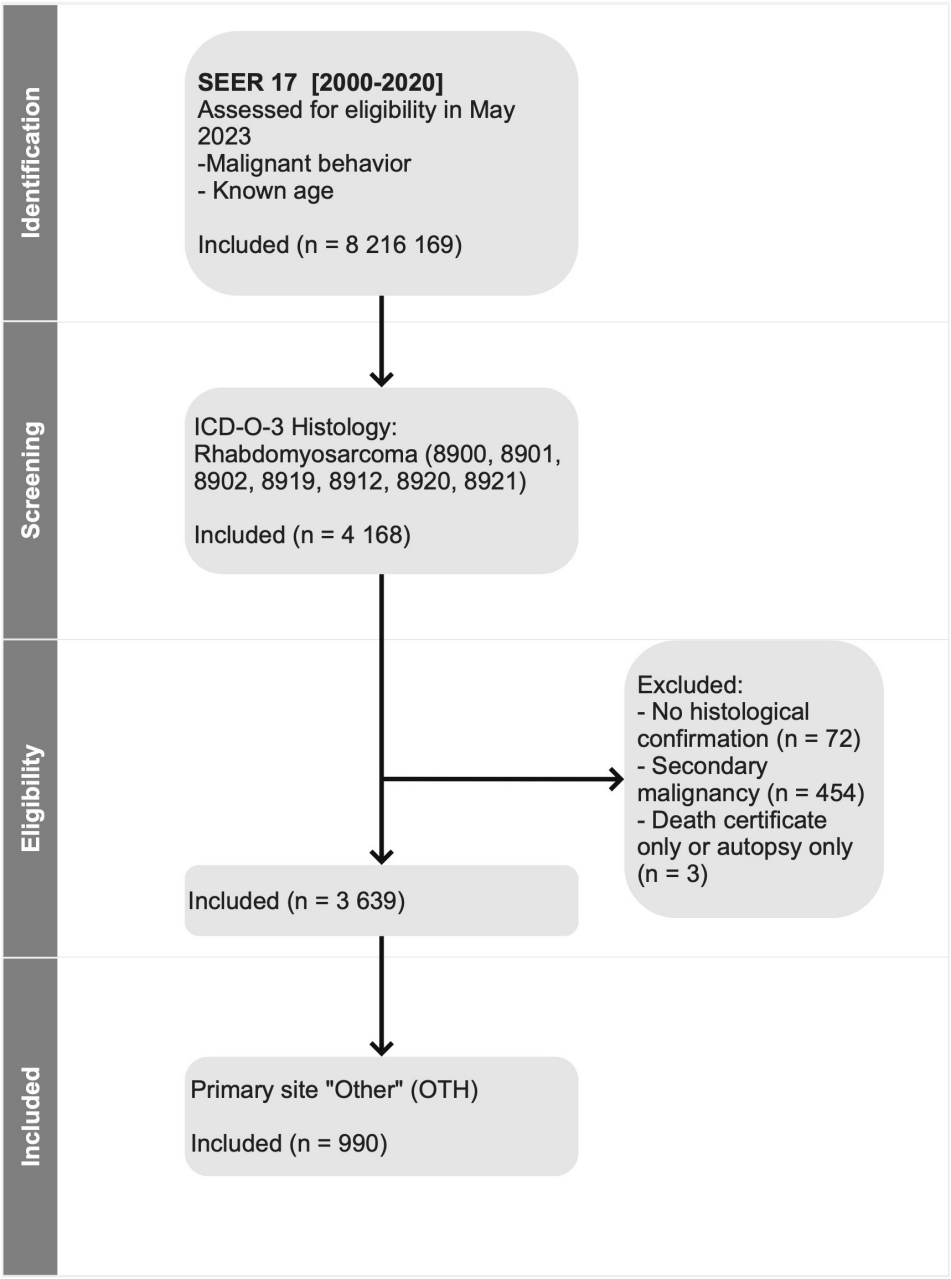
CONSORT diagram.

Patients were eligible for this analysis if the following criteria were fulfilled: (i) primary site: OTH, (ii) diagnostic confirmation: positive histology, (iii) sequence number: one primary only or first of two or more primaries. The data selection process is shown in Figure 1.

OTH was categorized in four different classifications based on localization, organ systems, anatomical structures, or outcome.

In the SEER database invasive neoplasms confined entirely to the organ of origin are staged as localized. The regional stage refers to a neoplasm that has extended beyond the limits of the organ of origin directly into surrounding organs or tissues, has spread to regional lymph nodes by way of the lymphatic system, or both. The distant stage is defined as a neoplasm that has spread to parts of the body remote from the primary tumor either by direct extension or by discontinuous metastasis to distant organs and tissues or via the lymphatic system to distant lymph nodes. Given the public availability of the SEER database and the anonymization of patient data, obtaining an ethics committee vote was deemed unnecessary, however, it was still procured from the Charité Berlin ethics committee. All available information was reviewed by the first author.

### 2.1 Statistical Methods

Statistics were calculated using SPSS 29. Overall survival (OS) and disease‐specific survival (DSS) were calculated using the Kaplan–Meier estimator [16]. To compare survival curves the log‐rank test was used [17]. For OS, vital status recode (study cutoff used) was used, for DSS the SEER cause‐specific death classification was assessed. OS and DSS were used as reported. For OS and DSS patients alive at the last follow‐up were censored, additionally for DSS patients without a disease‐specific death were censored. Confidence intervals (CI) for the Kaplan–Meier estimator were computed using Greenwoods Formula [18] and are stated at the 95% level. Univariates were analyzed using the Kaplan–Meier estimator and variables of interest were included in the Cox regression analysis. All variables included had a *p* < 0.001 in the univariate analysis. Multivariate analysis was conducted using the Cox proportional hazards regression method [19] to identify independent prognostic factors. Multiple Cox regression analyses were performed, including patient and tumor factors only, as well as analyses including therapy data. Cross‐classified tables were used to compare group differences and analyze subcategories and their distribution in age subgroups.

## 3 Results

### 3.1 Patient and Tumor Characteristics

The SEER 17 database 2000–2020 included data of 4168 patients with a diagnosis of RMS. A total of 72 patients were excluded due to missing positive histology, as a diagnostic confirmation. Furthermore, 454 patients with a previous recorded malignancy and three patients reported with death certificate only/autopsy only were excluded. Lastly, only data from patients whose primary site was defined as OTH were included. Consequently, data from 990 patients was included in the study. The gender distribution showed a slight male predominance with 56.9% male and 43.1% female patients. The dominant race recode was white (75.3%), followed by black (14.7%), with other being only 9.7%. Median age at diagnosis was 16 years (range 0–90+ years), with 53.1% of the patients being in childhood/adolescence (0–17 years). In the majority of cases, the histological type was Embryonal RMS, NOS (33.0%) or Alveolar RMS (25.5%). RMS, NOS made up 23.0%, Pleomorphic RMS, adult type 11.9%, Spindle cell RMS 5.0% and Mixed type RMS (including Mixed embryonal RMS and alveolar RMS) 1.6% of cases. Out of 674 patients with documented tumor size, tumor size was mainly ≥ 5 cm (84.4%), with the median tumor size being 9 cm (range 1 mm–52 cm). Within the site classification OTH, most tumors were in the pelvic area (site classification 1: 41.5%). The tumor stage in descending order was: distant (43.8%), regional (27.6%), localized (23.6%), unknown/unstaged/not reported (5.0%). Regional lymph nodes were generally not examined (77.8%), which is why regional lymph node involvement was mostly pathologically unknown (77.2%). Most patients were treated within 0 months (59.2%) to 1 month (25.3%) from the time of diagnosis. Chemotherapy was generally implemented (79.7%), while distribution of execution of radiotherapy (no: 50.1%, yes: 48.4%) and surgery of primary site (no: 50.5%, yes: 49.2%) was almost equal. Only a few patients underwent regional lymph node surgery (18.3%). More detailed information on the patient characteristics can be found in Tables 1 and 2.

**TABLE 1:** Univariate analysis of patients', tumor, and therapy characteristics.

	*n* (%)	5 years OS (95% CI)	*p*	5 years DSS (95% CI)	*p*
Gender					
Female	427 (43.1)	38.2 ± 4.9%	0.924	40.3 ± 5.1%	0.92
Male	563 (56.9)	38.4 ± 4.3%		41.0 ± 4.3%	
Race					
White	745 (75.3)	37.2 ± 3.7%	0.294	39.8 ± 3.7%	0.534
Black	146 (14.7)	43.9 ± 8.4%		45.4 ± 8.6%	
Other	96 (9.7)	38.7 ± 10.6%		40.2 ± 10.8%	
Age, years					
0–9	344 (34.7)	62.9 ± 5.5%	**< 0.001**	64.5 ± 5.5%	**< 0.001**
10–17	182 (18.4)	37.1 ± 7.5%		37.9 ± 7.5%	
18–39	148 (14.9)	30.2 ± 8.0%		31.8 ± 8.2%	
40–64	167 (16.9)	25.3 ± 7.1%		26.5 ± 7.3%	
≥ 65	149 (15.1)	5.6 ± 4.1%		11.1 ± 6.3%	
Histologic type					
RMS, NOS	228 (23.0)	20.8 ± 5.5%	**< 0.001**	23.7 ± 6.1%	**< 0.001**
Pleomorphic RMS, adult type	118 (11.9)	17.9 ± 7.6%		21.9 ± 8.6%	
Mixed type RMS	16 (1.6)	56.1 ± 26.7%		56.1 ± 26.7%	
Embryonal RMS, NOS	327 (33.0)	65.4 ± 5.5%		67.4 ± 5.5%	
Spindle cell RMS	49 (5.0)	41.6 ± 15.9%		45.7 ± 16.5%	
Alveolar RMS	252 (25.5)	28.1 ± 5.9%		28.8 ± 5.9%	
Size, cm					
< 5	105 (10.6)	59.8 ± 9.8%	**< 0.001**	60.6 ± 9.8%	**< 0.001**
5–10	301 (30.4)	44.3 ± 6.1%		48.0 ± 6.2%	
> 10	268 (27.1)	30.1 ± 5.9%		32.3 ± 6.2%	
Unknown	316 (31.9)	32.5 ± 5.3%		34.0 ± 5.5%	
Site classification 1					
Abdomen and retroperitoneum	286 (28.9)	30.7 ± 5.7%	**< 0.001**	32.4 ± 5.9%	**< 0.001**
Thorax	220 (22.2)	28.7 ± 6.3%		33.0 ± 6.7%	
Pelvis	411 (41.5)	49.2 ± 5.1%		50.6 ± 5.3%	
Dorsum	3 (0.3)	0%		0%	
Other	70 (7.1)	37.5 ± 12.9%		40.9 ± 13.3%	
Site classification 2					
Breast	13 (1.3)	67.7 ± 26.3%	**< 0.001**	67.7 ± 26.3%	**< 0.001**
GI‐Tract (incl. rectum and anus)	12 (1.2)	41.7 ± 27.8%		41.7 ± 27.8%	
Kidney and adrenal Gland	14 (1.4)	23.8 ± 25.3%		23.8 ± 25.3%	
Liver and biliary	22 (2.2)	45.6 ± 21.8%		45.6 ± 21.8%	
Lung	24 (2.4)	0%		0%	
Mediastinum and pleura	41 (4.1)	20.2 ± 12.9%		23.1 ± 13.9%	
Peritoneum and retroperitoneum	82 (8.3)	32.4 ± 10.4%		37.2 ± 11.4%	
Pancreas	4 (0.4)	0%		0%	
Pelvis other	4 (0.4)	50.0 ± 49.0%		50.0 ± 49.0%	
Skin of trunk	1 (0.1)	0%		0%	
Spinal	4 (0.4)	25.0 ± 42.5%		25.0 ± 42.5	
Tissue abdomen (incl. urachus)	159 (16.1)	29.2 ± 7.6%		30.3 ± 7.8%	
Tissue pelvis	402 (40.6)	49.0 ± 5.1%		50.5 ± 5.3%	
Tissue thorax	140 (14.1)	32.1 ± 8.0%		36.8 ± 8.6%	
Tissue trunk	68 (6.9)	37.5 ± 13.1%		41.3 ± 13.3%	
Site classification 3					
Parenchymatous organs	50 (5.1)	10.8 ± 9.2%	**< 0.001**	11.8 ± 10.0%	**< 0.001**
Hollow organs	29 (2.9)	43.5 ± 19.0%		45.3 ± 19.6%	
Cavities	117 (11.8)	28.2 ± 8.4%		32.0 ± 9.2%	
Tissue abdomen	158 (16.0)	29.1 ± 7.6%		30.2 ± 7.8%	
Tissue pelvis	402 (40.6)	49.0 ± 5.1%		50.5 ± 5.3%	
Tissue thorax	140 (14.1)	32.1 ± 8.0%		36.8 ± 8.6%	
Tissue trunk	82 (8.3)	42.4 ± 12.0%		45.3 ± 12.2%	
Other	12 (1.2)	50.0 ± 28.2%		50.0 ± 28.2%	
Site classification 4					
OTH favorable	29 (2.9)	69.7 ± 17.6%	**< 0.001**	69.7 ± 17.6%	**< 0.001**
Heart	7 (0.7)	28.6 ± 33.5%		33.3 ± 37.6%	
Intestines	7 (0.7)	28.6 ± 33.5%		28.6 ± 33.5%	
Kidney	13 (1.3)	25.6 ± 27.1%		25.6 ± 27.1%	
Liver	10 (1.0)	20.0 ± 24.7%		20.0 ± 24.7%	
Lung and pleura	29 (2.9)	3.6 ± 6.9%		4.6 ± 8.6%	
Mediastinum	30 (3.0)	17.0 ± 14.7%		19.9 ± 16.1%	
Pancreas	4 (0.4)	0.0 ± 0.0%		0.0 ± 0.0%	
Pelvis and vertebral	6 (0.6)	16.7 ± 29.8%		16.7 ± 29.8%	
Peritoneum	12 (1.2)	33.3 ± 26.7%		33.3 ± 26.7%	
Retroperitoneum	71 (7.2)	31.6 ± 11.2%		37.0 ± 12.4%	
Tissue abdomen	159 (16.1)	29.2 ± 7.6%		30.3 ± 7.8%	
Tissue pelvis	403 (40.7)	49.2 ± 5.1%		50.6 ± 5.3%	
Tissue thorax	140 (14.1)	32.1 ± 8.0%		36.8 ± 8.6%	
Tissue trunk	70 (7.1)	37.5 ± 12.9%		40.9 ± 13.3%	
Stage					
Distant site(s)/node(s) involved	434 (43.8)	18.6 ± 3.9%	**< 0.001**	19.9 ± 4.1%	**< 0.001**
Regional by direct extension only	128 (12.9)	45.3 ± 8.8%		46.3 ± 8.8%	
Regional lymph nodes involved only	34 (3.4)	59.7 ± 17.1%		61.6 ± 17.2%	
Regional by both direct extension and lymph node involvement	27 (2.7)	59.3 ± 18.6%		59.3 ± 18.6%	
Regional	84 (8.5)	52.8 ± 12.9%		55.4 ± 13.3%	
Localized only	234 (23.6)	61.8 ± 6.7%		66.5 ± 6.5%	
Unstaged	49 (5.0)	37.2 ± 13.9%		43.0 ± 14.9%	
Regional lymph nodes examined					
No	721 (72.8)	32.0 ± 3.7%	**< 0.001**	34.5 ± 3.7%	**< 0.001**
Yes	228 (23.0)	57.1 ± 6.9%		58.4 ± 6.9%	
Unknown	41 (4.1)	45.2 ± 16.1%		47.8 ± 16.5%	
Regional lymph nodes positive pathologically					
N0	133 (13.4)	68.5 ± 8.4%	**< 0.001**	70.5 ± 8.2%	**< 0.001**
N1	93 (9.4)	40.9 ± 10.4%		40.9 ± 10.4%	
Nx	764 (77.2)	32.8 ± 3.5%		35.4 ± 3.7%	
Time from diagnosis to treatment, months					
0	586 (59.2)	46.5 ± 4.3%	**< 0.001**	48.2 ± 4.3%	**< 0.001**
1	250 (25.3)	30.0 ± 6.3%		33.1 ± 6.5%	
2	48 (4.8)	24.4 ± 12.9%		26.8 ± 13.7%	
3	8 (0.8)	25.0 ± 30.0%		25.0 ± 30.0%	
4	3 (0.3)	50.0 ± 69.4%		50.0 ± 69.4%	
5	2 (0.2)	50.0 ± 69.4%		50.0 ± 69.4%	
6	3 (0.3)	33.3 ± 53.3%		33.3 ± 53.3%	
7	1 (0.1)	0%		0%	
Not reported	89 (9.0)	15.4 ± 8.0%		19.1 ± 9.4%	
Chemotherapy					
No/Unknown	201 (20.3)	23.9 ± 6.3%	**< 0.001**	30.1 ± 7.1%	**< 0.001**
Yes	789 (79.7)	42.1 ± 3.7%		43.6 ± 3.7%	
Radiotherapy					
No/Unknown	496 (50.1)	29.1 ± 4.3%	**< 0.001**	32.5 ± 4.5%	**< 0.001**
Yes	479 (48.4)	47.9 ± 4.7%		49.1 ± 4.7%	
Radiation recommended, unknown if administered	15 (1.5)	31.4 ± 26.7%		31.4 ± 26.7%	
Surgery primary site					
No	500 (50.5)	22.5 ± 3.9%	**< 0.001**	24.6 ± 4.1%	**< 0.001**
Yes	487 (49.2)	54.5 ± 4.7%		56.6 ± 4.7%	
Unknown	3 (0.3)	33.3 ± 53.3%		33.3 ± 53.3%	
Surgery regional lymph nodes					
No	774 (78.2)	32.9 ± 3.5%	**< 0.001**	35.3 ± 3.7%	**< 0.001**
Yes	181 (18.3)	60.4 ± 7.6%		61.7 ± 7.6%	
Unknown/Not applicable	27 (2.7)	43.0 ± 19.0%		46.8 ± 20.0%	
Not reported	8 (0.8)	50.0 ± 34.7%		50.0 ± 34.7%	

*Note:* Bold values indicate statistical significance *p* < 0.05.

**TABLE 2:** Cross‐classified table for age groups.

	0–9 years	10–17 years	18–39 years	40–64 years	≥ 65 years	Total *n* = (%)
Age group	344 (34.7)	182 (18.4)	148 (14.9)	167 (16.9)	149 (15.1)	990 (100)
*Site classifications*						
1						
Abdomen and retroperitoneum	116 (40.6)	31 (10.8)	29 (10.1)	59 (20.6)	51 (17.8)	286 (28.9)
Thorax	52 (23.6)	38 (17.3)	34 (15.5)	45 (20.5)	51 (23.2)	220 (22.2)
Pelvis	158 (38.4)	100 (24.3)	79 (19.2)	42 (10.2)	32 (7.8)	411 (41.5)
Dorsum	1 (33.3)	0 (0.0)	0 (0.0)	2 (66.7)	0 (0.0)	3 (0.3)
Other	17 (24.3)	13 (18.6)	6 (8.6)	19 (27.1)	15 (21.4)	70 (7.1)
2						
Breast	0 (0.0)	3 (23.1)	4 (30.8)	5 (38.5)	1 (7.7)	13 (1.3)
GI‐tract (incl. rectum and anus)	3 (25.0)	1 (8.3)	2 (16.7)	4 (33.3)	2 (16.7)	12 (1.2)
Kidney and adrenal gland	2 (14.3)	2 (14.3)	2 (14.3)	4 (28.6)	4 (28.6)	14 (1.4)
Liver and biliary	16 (72.7)	1 (4.5)	0 (0.0)	2 (9.1)	3 (13.6)	22 (2.2)
Lung	3 (12.5)	1 (4.2)	1 (4.2)	4 (16.7)	15 (62.5)	24 (2.4)
Mediastinum and pleura	7 (17.1)	5 (12.2)	12 (29.3)	8 (19.5)	9 (22.0)	41 (4.1)
Peritoneum and retroperitoneum	32 (39.0)	8 (9.8)	4 (4.9)	21 (25.6)	17 (20.7)	82 (8.3)
Pancreas	1 (25.0)	1 (25.0)	0 (0.0)	2 (50.0)	0 (0.0)	4 (0.4)
Pelvis other	1 (25.0)	0 (0.0)	2 (50.0)	1 (25.0)	0 (0.0)	4 (0.4)
Skin of trunk	0 (0.0)	0 (0.0)	0 (0.0)	1 (100.0)	0 (0.0)	1 (0.1)
Spinal	1 (25.0)	0 (0.0)	0 (0.0)	3 (75.0)	0 (0.0)	4 (0.4)
Tissue abdomen (incl. urachus)	64 (40.3)	19 (11.9)	22 (13.8)	28 (17.6)	26 (16.4)	159 (16.1)
Tissue pelvis	155 (38.6)	99 (24.6)	76 (18.9)	40 (10.0)	32 (8.0)	402 (40.6)
Tissue thorax	42 (30.0)	29 (20.7)	17 (12.1)	27 (19.3)	25 (17.9)	140 (14.1)
Tissue trunk	17 (25.0)	13 (19.1)	6 (8.8)	17 (25.0)	15 (22.1)	68 (6.9)
3						
Parenchymatous organs	11 (22.0)	3 (6.0)	3 (6.0)	11 (22.0)	22 (44.0)	50 (5.1)
Hollow organs	13 (44.8)	2 (6.9)	3 (10.3)	6 (20.7)	5 (17.2)	29 (2.9)
Cavities	39 (33.3)	13 (11.1)	14 (12.0)	28 (23.9)	23 (19.7)	117 (11.8)
Tissue abdomen	63 (39.9)	19 (12.0)	22 (13.9)	28 (17.7)	26 (16.5)	158 (16.0)
Tissue pelvis	155 (38.6)	99 (24.6)	76 (18.9)	40 (10.0)	32 (8.0)	402 (40.6)
Tissue thorax	42 (30.0)	29 (20.7)	17 (12.1)	27 (19.3)	25 (17.9)	140 (14.1)
Tissue trunk	17 (20.7)	16 (19.5)	10 (12.2)	23 (28.0)	16 (19.5)	82 (8.3)
Other	4 (33.3)	1 (8.3)	3 (25.0)	4 (33.3)	0 (0.0)	12 (1.2)
4						
OTH favorable	13 (44.8)	5 (17.2)	5 (17.2)	5 (17.2)	1 (3.4)	29 (2.9)
Heart	0 (0.0)	0 (0.0)	2 (28.6)	2 (28.6)	3 (42.9)	7 (0.7)
Intestines	1 (14.3)	0 (0.0)	1 (14.3)	4 (57.1)	1 (14.3)	7 (0.7)
Kidney	2 (15.4)	2 (15.4)	2 (15.4)	3 (23.1)	4 (30.8)	13 (1.3)
Liver	5 (50.0)	0 (0.0)	0 (0.0)	2 (20.0)	3 (30.0)	10 (1.0)
Lung and pleura	5 (17.2)	1 (3.4)	2 (6.9)	5 (17.2)	16 (55.2)	29 (2.9)
Mediastinum	5 (16.7)	5 (16.7)	9 (30.0)	5 (16.7)	6 (20.0)	30 (3.0)
Pancreas	1 (25.0)	1 (25.0)	0 (0.0)	2 (50.0)	0 (0.0)	4 (0.4)
Pelvis and vertebral	1 (16.7)	0 (0.0)	2 (33.3)	3 (50.0)	0 (0.0)	6 (0.6)
Peritoneum	6 (50.0)	0 (0.0)	0 (0.0)	4 (33.3)	2 (16.7)	12 (1.2)
Retroperitoneum	26 (36.6)	8 (11.3)	4 (5.6)	18 (25.4)	15 (21.1)	71 (7.2)
Tissue abdomen	64 (40.3)	19 (11.9)	22 (13.8)	28 (17.6)	26 (16.4)	159 (16.1)
Tissue pelvis	156 (38.7)	99 (24.6)	76 (18.9)	40 (9.9)	32 (7.9)	403 (40.7)
Tissue thorax	42 (30.0)	29 (20.7)	17 (12.1)	27 (19.3)	25 (17.9)	140 (14.1)
Tissue trunk	17 (24.3)	13 (18.6)	6 (8.6)	19 (27.1)	15 (21.4)	70 (7.1)
Size, cm						
< 5	52 (49.5)	14 (13.3)	10 (9.5)	15 (14.3)	14 (13.3)	105 (10.6)
5–10	115 (38.2)	66 (21.9)	49 (16.3)	38 (12.6)	33 (11.0)	301 (30.4)
> 10	76 (28.4)	33 (12.3)	42 (15.7)	59 (22.0)	58 (21.6)	268 (27.1)
Unknown	101 (32.0)	69 (21.8)	47 (14.9)	55 (17.4)	44 (13.9)	316 (31.9)
Stage						
Localized	105 (44.9)	26 (11.1)	35 (15.0)	36 (15.4)	32 (13.7)	234 (23.6)
Regional	116 (42.5)	39 (14.3)	33 (12.1)	47 (17.2)	38 (13.9)	273 (27.6)
Distant	111 (25.6)	110 (25.3)	74 (17.1)	71 (16.4)	68 (15.7)	434 (43.8)
Unstaged	12 (24.5)	7 (14.3)	6 (12.2)	13 (26.5)	11 (22.4)	49 (4.9)
Histologic type						
RMS, NOS	41 (18.0)	22 (9.6)	26 (11.4)	68 (29.8)	71 (31.1)	228 (23.0)
Pleomorphic RMS, adult type	2 (1.7)	0 (0.0)	14 (11.9)	54 (45.8)	48 (40.7)	118 (11.9)
Mixed type RMS	10 (62.5)	1 (6.3)	2 (12.5)	1 (6.3)	2 (12.5)	16 (1.6)
Embryonal RMS, NOS	199 (60.9)	50 (15.3)	42 (12.8)	24 (7.3)	12 (3.7)	327 (33.0)
Spindle cell RMS	15 (30.6)	3 (6.1)	13 (26.5)	9 (18.4)	9 (18.4)	49 (4.9)
Alveolar RMS	77 (30.6)	106 (42.1)	51 (20.2)	11 (4.4)	7 (2.8)	252 (25.5)
Regional lymph nodes examined pathologically						
No	239 (33.1)	115 (16.0)	111 (15.4)	125 (17.3)	131 (18.2)	721 (72.8)
Yes	88 (38.6)	60 (26.3)	30 (13.2)	36 (15.8)	14 (6.1)	228 (23.0)
Unknown	17 (41.5)	7 (17.1)	7 (17.1)	6 (14.6)	4 (9.8)	41 (4.1)
Regional lymph nodes positive pathologically						
N0	65 (48.9)	21 (15.8)	14 (10.5)	23 (17.3)	10 (7.5)	133 (13.4)
N1	23 (24.7)	38 (40.9)	15 (16.1)	13 (14.0)	4 (4.3)	93 (9.4)
Nx	256 (33.5)	123 (16.1)	119 (15.6)	131 (17.1)	135 (17.7)	764 (77.2)
Surgery primary site						
No	147 (29.4)	110 (22.0)	86 (17.2)	71 (14.2)	86 (17.2)	500 (50.5)
Yes	196 (40.2)	72 (14.8)	61 (12.5)	96 (19.7)	62 (12.7)	487 (49.2)
Unknown	1 (33.3)	0 (0.0)	1 (33.3)	0 (0.0)	1 (33.3)	3 (0.3)
Surgery regional lymph nodes						
No	259 (3350)	132 (17.1)	115 (14.9)	132 (17.1)	136 (17.6)	774 (78.2)
Yes	79 (43.6)	43 (23.8)	24 (13.3)	27 (14.9)	8 (4.4)	181 (18.3)
Unknown/Not applicable	5 (18.5)	6 (22.2)	5 (18.5)	7 (25.9)	4 (14.8)	27 (2.7)
Not reported	1 (12.5)	1 (12.5)	4 (50.0)	1 (12.5)	1 (12.5)	8 (0.8)
Radiation						
No	121 (24.4)	64 (12.9)	91 (18.3)	107 (21.6)	113 (22.8)	496 (50.1)
Yes	221 (46.1)	113 (23.6)	51 (10.6)	60 (12.5)	34 (7.1)	479 (48.4)
Unknown	2 (13.3)	5 (33.3)	6 (40.0)	0 (0.0)	2 (13.3)	15 (1.5)
Chemotherapy						
No/Unknown	15 (7.5)	8 (4.0)	23 (11.4)	63 (31.3)	92 (45.8)	201 (20.3)
Yes	329 (41.7)	174 (22.1)	125 (15.8)	104 (13.2)	57 (7.2)	789 (79.7)

### 3.2 Outcome

**FIGURE 2:**
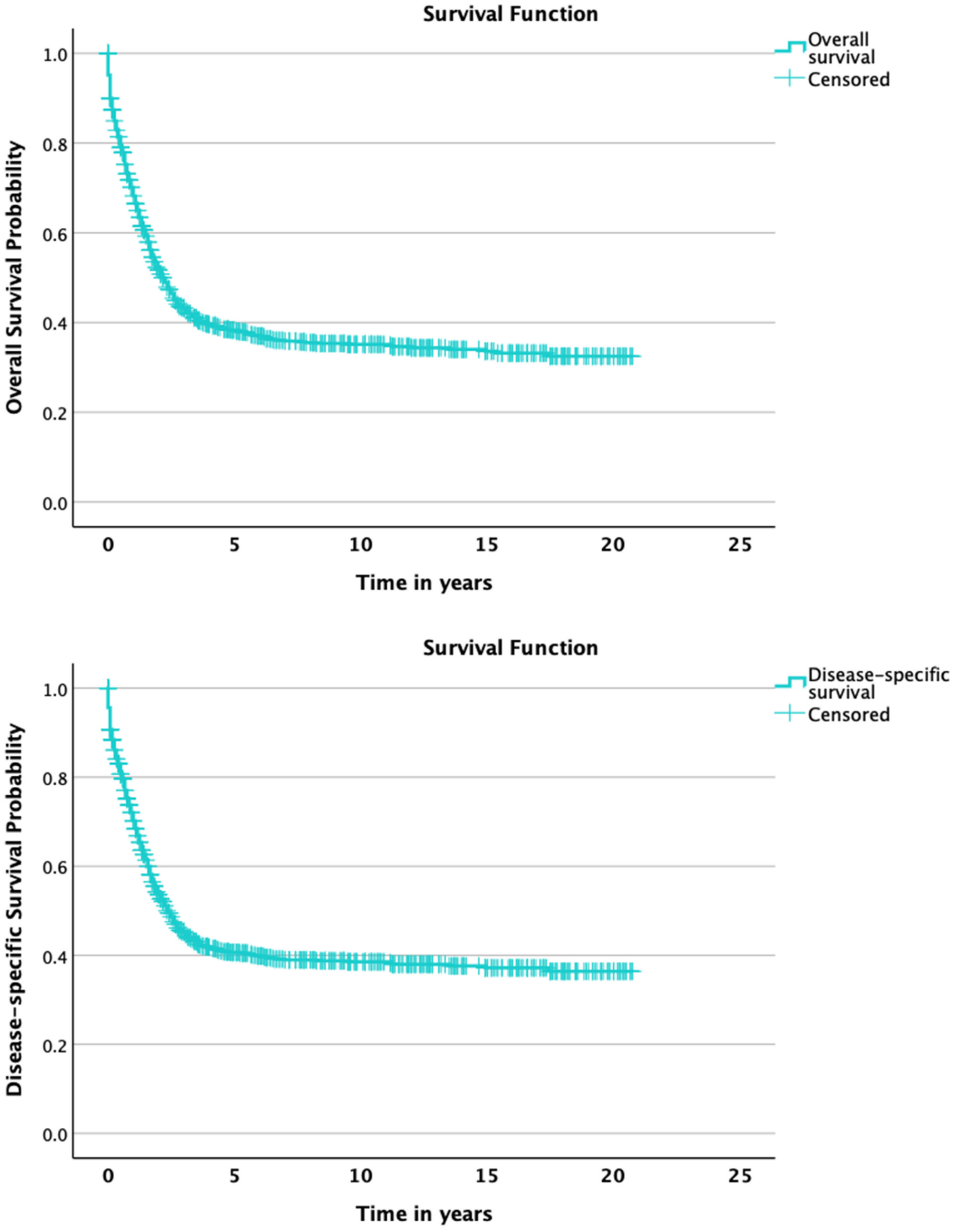
Survival.

**FIGURE 3:**
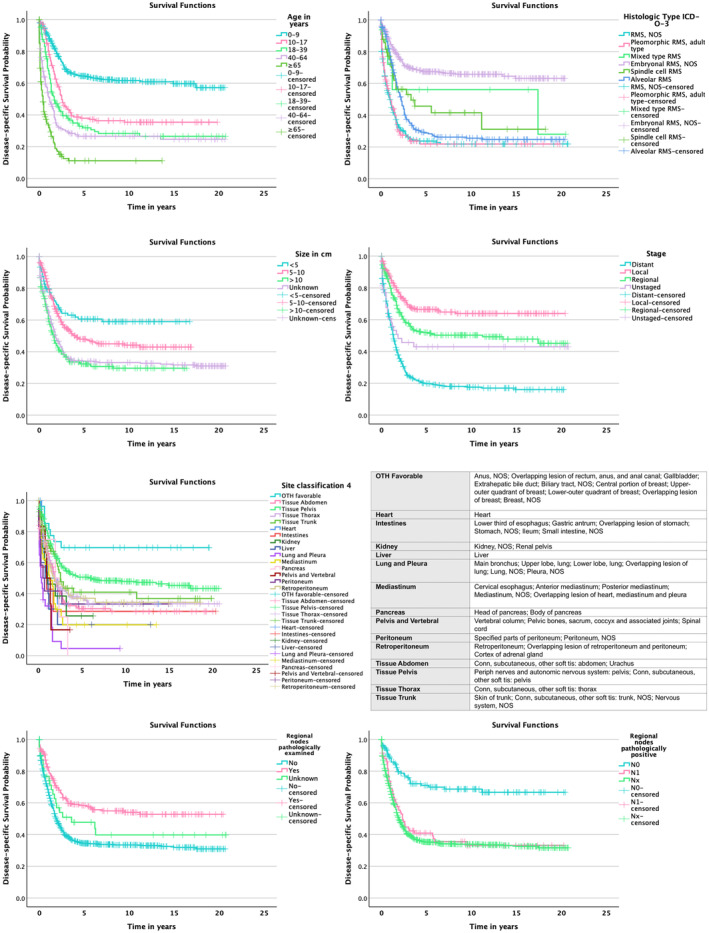
DSS variables.

Results indicated that 3‐, 5‐, and 10‐year OS probability was 45.4% ± 3.332 (95% CI), 40.7 ± 3.332, and 38.6% ± 3.332, respectively, and 3‐, 5‐, and 10‐year‐DSS probability was 43.3% ± 3.136 (95% CI), 38.3% ± 3.136, and 35.1% ± 3.332, respectively (Figures 2 and 3, Figure A1).

In total, 40.1% of patients were alive at the cutoff date with a median follow‐up of 9.96 years (range 0–20.75 years) for survivors. Of 593 patients, it was reported that the majority died of their disease (92.4%), with a median time to death of disease of 1.58 years (range 0–17.42 years). Further details regarding the cause of death can be found in Table A1.

### 3.3 Prognostic Factors

Univariate analysis of predictors of survival is shown in Table 1. In the analysis, gender and race of patient were not predictive for survival. Predictive for OS and DSS were age, histologic type, size, and stage. Older age, larger tumor size, and progressed stage correlated with adverse survival rates. The cross‐classified table for age groups (Table 2) shows higher numbers of small tumor sizes, localized or regional tumors, favorable histological type (Embryonal RMS), and performance of regional lymph node examination and therapeutic interventions, along with fewer cases of pathologically positive regional lymph nodes, within the pediatric age groups (0–9 and 10–17 years).

### 3.4 Cox Regression Analysis

To establish independent prognostic significance of primary tumor site, multiple multivariate models were performed. Variables included in the main analysis were age, histological type, tumor site classification 4, tumor size, and stage. Other multivariate analyses performed included regional lymph node examination, regional lymph node involvement, surgery of primary site, radiotherapy, chemotherapy, and surgery of regional lymph nodes. Variables independently associated with OS and DSS in the main analysis were age, histological type, site, and stage. Partially only certain subgroups within the variables were significant or showed borderline significance. Further information can be found in Table 3.

**TABLE 3:** Multivariate analysis of patients' and tumor characteristics.

	Overall survival				Disease specific survival			
	Hazard ratio	95% CI, lower	95% CI, upper	*p*	Hazard ratio	95% CI, lower	95% CI, upper	*p*
Age, years								
0–9	1.0			**< 0.001**	1.0			**< 0.001**
10–17	1.279	0.972	1.684	0.079	1.316	0.991	1.748	0.058
18–39	1.894	1.430	2.507	**< 0.001**	1.972	1.477	2.633	**< 0.001**
40–64	3.072	2.283	4.135	**< 0.001**	3.109	2.281	4.239	**< 0.001**
≥ 65	6.086	4.465	8.296	**< 0.001**	5.452	3.923	7.577	**< 0.001**
Histological type								
Embryonal RMS, NOS	1.0			**< 0.001**	1.0			**< 0.001**
Mixed type RMS	1.494	0.690	3.238	0.309	1.641	0.755	3.568	0.212
Spindle cell RMS	1.630	1.049	2.531	**0.030**	1.635	1.027	2.602	**0.038**
Alveolar RMS	1.975	1.523	2.560	**< 0.001**	2.026	1.548	2.652	**< 0.001**
Pleomorphic adult type	1.731	1.237	2.420	**0.001**	1.734	1.216	2.471	**0.002**
RMS, NOS	2.080	1.578	2.741	**< 0.001**	2.056	1.537	2.751	**< 0.001**
Site classification 4								
OTH favorable	1.0			**0.005**	1.0			**0.004**
Tissue abdomen	2.493	1.200		**0.014**	2.230	1.071	4.642	**0.032**
Tissue pelvis	1.882	0.923	5.179	0.082	1.689	0.828	3.446	0.150
Tissue thorax	2.319	1.119	3.837	**0.024**	1.983	0.953	4.127	0.067
Tissue trunk	1.703	0.789	4.806	0.175	1.556	0.716	3.379	0.264
Heart	1.908	0.615	3.677	0.264	1.527	0.452	5.155	0.495
Intestines	2.658	0.846	5.927	0.094	2.784	0.885	8.755	0.080
Kidney	1.554	0.603	8.359	0.361	1.416	0.537	3.730	0.482
Liver	5.225	1.911	4.001	**0.001**	5.901	2.157	16.146	**< 0.001**
Lung and pleura	3.844	1.706	14.28	**0.001**	3.575	1.573	8.126	**0.002**
Mediastinum	3.066	1.357	8.660	**0.007**	2.740	1.207	6.220	**0.016**
Pancreas	1.949	0.575	6.928	0.284	1.730	0.510	5.873	0.379
Pelvis and vertebral	3.077	0.981	6.608	0.054	2.851	0.906	8.974	0.073
Peritoneum	1.606	0.587	4.395	0.356	1.593	0.581	4.367	0.366
Retroperitoneum	2.357	1.101	5.048	**0.027**	1.835	0.846	3.982	0.124
Size, cm								
< 5	1.0		1.593	0.091	1.0			0.050
5–10	1.120	0.788	1.914	0.526	1.029	0.712	1.486	0.878
> 10	1.341	0.940	1.969	0.106	1.310	0.905	1.897	0.153
Unknown/Not reported	1.392	0.985		0.061	1.355	0.945	1.942	0.098
Stage								
Localized	1.0			**< 0.001**	1.0			**< 0.001**
Regional	1.333	1.006	1.766	**0.045**	1.501	1.110	2.031	**0.008**
Distant	3.200	2.466	4.151	**< 0.001**	3.615	2.729	4.789	**< 0.001**
Unstaged	1.643	1.057	2.555	**0.027**	1.495	0.915	2.443	0.108
Age, years								
0–9	1.0			**< 0.001**	1.0			**< 0.001**
10–17	1.281	0.972	1.687	0.079	1.318	0.992	1.751	0.057
18–39	1.843	1.391	2.443	**< 0.001**	1.920	1.436	2.566	**< 0.001**
40–64	3.064	2.276	4.126	**< 0.001**	3.116	2.286	4.249	**< 0.001**
≥ 65	5.902	4.330	8.045	**< 0.001**	5.315	3.825	7.387	**< 0.001**
Histological type								
Embryonal RMS, NOS	1.0			**< 0.001**	1.0			**< 0.001**
Mixed type RMS	1.422	0.655	3.084	0.373	1.568	0.720	3.413	0.257
Spindle cell RMS	1.633	1.050	2.538	**0.029**	1.637	1.028	2.609	**0.038**
Alveolar RMS	2.042	1.572	2.652	**< 0.001**	2.093	1.596	2.745	**< 0.001**
Pleomorphic adult type	1.730	1.238	2.419	**0.001**	1.731	1.215	2.466	**0.002**
RMS, NOS	2.055	1.560	2.707	**< 0.001**	2.028	1.517	2.712	**< 0.001**
Site classification 4								
OTH favorable	1.0			**0.014**	1.0			**0.009**
Tissue abdomen	2.131	1.018	4.458	**0.045**	1.925	0.918	4.035	0.083
Tissue pelvis	1.659	0.809	3.399	0.167	1.504	0.734	3.084	0.265
Tissue thorax	1.968	0.942	4.113	0.072	1.698	0.809	3.563	0.161
Tissue trunk	1.460	0.671	3.176	0.340	1.347	0.615	2.947	0.456
Heart	1.583	0.506	4.951	0.430	1.283	0.377	4.359	0.690
Intestines	2.163	0.677	6.911	0.193	2.248	0.703	7.187	0.172
Kidney	1.370	0.529	3.547	0.516	1.270	0.480	3.360	0.631
Liver	4.354	1.580	12.003	**0.004**	4.980	1.805	13.737	**0.002**
Lung and pleura	3.335	1.473	7.550	**0.004**	3.127	1.369	7.143	**0.007**
Mediastinum	2.730	1.204	6.190	**0.016**	2.469	1.084	5.623	**0.031**
Pancreas	1.822	0.538	6.171	0.335	1.639	0.484	5.553	0.428
Pelvis and vertebral	2.580	0.815	8.169	0.107	2.409	0.758	7.653	0.136
Peritoneum	1.392	0.505	3.833	0.523	1.391	0.504	3.839	0.524
Retroperitoneum	2.012	0.933	4.342	0.075	1.579	0.722	3.452	0.252
Size, cm								
< 5	1.0			0.121	1.0			0.069
5–10	1.122	0.789	1.596	0.520	1.028	0.711	1.485	0.884
> 10	1.339	0.938	1.910	0.108	1.305	0.901	1.889	0.159
Unknown/Not reported	1.370	0.968	1.938	0.075	1.329	0.927	1.906	0.122
Stage								
Localized	1.0			**< 0.001**	1.0			**< 0.001**
Regional	1.398	1.053	1.855	**0.021**	1.574	1.162	2.133	**0.003**
Distant	3.190	2.460	4.138	**< 0.001**	3.603	2.720	4.771	**< 0.001**
Unstaged	1.668	1.052	2.646	**0.030**	1.499	0.899	2.499	0.121
Regional lymph nodes examined pathologically								
Yes	1.0			**0.026**	1.0			**0.040**
No	1.377	1.091	1.737	**0.007**	1.364	1.073	1.734	**0.011**
Unknown	1.239	0.748	2.053	0.405	1.292	0.766	2.177	0.336
Age, years								
0–9	1.0			**< 0.001**	1.0			**< 0.001**
10–17	1.238	0.939	1.633	0.130	1.271	0.956	1.690	0.100
18–39	1.808	1.363	2.397	**< 0.001**	1.881	1.407	2.516	**< 0.001**
40–64	3.039	2.260	4.087	**< 0.001**	3.084	2.264	4.200	**< 0.001**
≥ 65	5.778	4.245	7.865	**< 0.001**	5.186	3.739	7.195	**< 0.001**
Histological type								
Embryonal RMS, NOS	1.0			**< 0.001**	1.0			**< 0.001**
Mixed type RMS	1.396	0.644	3.027	0.398	1.522	0.700	3.311	0.290
Spindle cell RMS	1.677	1.077	2.610	**0.022**	1.684	1.056	2.687	**0.029**
Alveolar RMS	1.991	1.531	2.589	**< 0.001**	2.029	1.545	2.664	**< 0.001**
Pleomorphic adult type	1.747	1.250	2.442	**0.001**	1.745	1.225	2.485	**0.002**
RMS, NOS	2.083	1.581	2.743	**< 0.001**	2.052	1.536	2.743	**< 0.001**
Site classification 4								
OTH favorable	1.0			**0.006**	1.0			**0.004**
Tissue abdomen	2.065	0.987	4.320	0.054	1.874	0.894	3.928	0.096
Tissue pelvis	1.546	0.754	3.167	0.234	1.403	0.684	2.875	0.355
Tissue thorax	1.875	0.897	3.919	0.095	1.624	0.774	3.407	0.199
Tissue trunk	1.387	0.637	3.019	0.410	1.283	0.586	2.810	0.533
Heart	1.503	0.481	4.698	0.484	1.217	0.358	4.132	0.753
Intestines	2.023	0.637	6.430	0.232	2.131	0.670	6.780	0.200
Kidney	1.331	0.514	3.448	0.556	1.245	0.470	3.300	0.659
Liver	4.152	1.508	11.434	**0.006**	4.703	1.706	12.965	**0.003**
Lung and pleura	3.288	1.452	7.445	**0.004**	3.098	1.356	7.080	**0.007**
Mediastinum	2.755	1.215	6.245	**0.015**	2.525	1.109	5.750	**0.027**
Pancreas	1.536	0.451	5.233	0.492	1.370	0.402	4.671	0.615
Pelvis and vertebral	2.421	0.762	7.689	0.134	2.261	0.709	7.213	0.168
Peritoneum	1.316	0.478	3.619	0.595	1.321	0.479	3.641	0.590
Retroperitoneum	1.976	0.916	4.265	0.083	1.557	0.712	3.404	0.267
Size, cm								
< 5	1.0			0.127	1.0			0.069
5–10	1.114	0.784	1.584	0.547	1.018	0.705	1.471	0.922
> 10	1.328	0.931	1.894	0.117	1.294	0.894	1.873	0.172
Unknown/Not reported	1.360	0.962	1.922	0.082	1.320	0.921	1.891	0.131
Stage								
Localized	1.0			**< 0.001**	1.0			**< 0.001**
Regional	1.354	1.019	1.800	**0.037**	1.520	1.120	2.063	**0.007**
Distant	3.054	2.349	3.969	**< 0.001**	3.431	2.585	4.554	**< 0.001**
Unstaged	1.558	1.003	2.421	**0.049**	1.414	0.866	2.308	0.166
Regional lymph nodes positive pathologically								
N0	1.0			**0.002**	1.0			**0.002**
N1	1.819	1.194	2.773	**0.005**	1.930	1.246	2.990	**0.003**
Nx	1.812	1.304	2.517	**< 0.001**	1.881	1.326	2.668	**< 0.001**

*Note:* Bold values indicate statistical significance *p* < 0.05.

### 3.5 Site Classification

Site of primary tumor was performed in different classifications. All site classifications were predictive for OS and DSS within univariate analysis with a *p* < 0.001. The first classification performed differentiates the localization in the sites: abdomen and retroperitoneum, thorax, pelvis, dorsum, and other. The groups pelvis and other correlated with a better outcome in this classification, whereas the group dorsum correlated with the worst outcome in both OS and DSS. The second classification performed differentiates the site in organ systems and anatomical structures. Breast was shown to be the site with the best OS and DSS rates within this site classification. Lung, pancreas, and skin of trunk showed the worst OS and DSS rates. The third classification divides the site of primary tumor in parenchymatous organs, hollow organs, cavities, tissue thorax, tissue abdomen, tissue pelvis, tissue trunk, torso, and other. Within this classification of sites, the site of primary tumor within the group torso showed the best survival rates, while the group parenchymatous organs had the worst outcome. The fourth site classification performed distinguishes between organ systems and anatomical structures, with the division and grouping of categories being based on outcomes in univariate and multivariate analyses. Hence, a favorable subgroup was created, and the lungs and pleura, as well as the pelvical structures and vertebral area, were each combined into their own subgroup. The OTH favorable subgroup consists of the breast, the perianal region, and biliary structures. This subgroup was predictably shown to correlate with the best outcome, while the subgroups lung and pleura, the subgroup mediastinum and the subgroup liver correlated adversely with OS and DSS. More information regarding outcomes can be found in Tables 1 and 3. More details regarding the granular site classifications and subgroups can be found in Table A2.

### 3.6 Regional Lymph Nodes

As shown in Table 1, examination of regional lymph nodes, no nodal involvement, and regional lymph node surgery were positively associated with OS and DSS in the univariate analysis. In the multivariate analysis, regional lymph node examination and pathologically positive regional lymph nodes were independently associated with OS and DSS. Furthermore, the significance of other variables within the multivariate analysis was impacted through including the variables regional lymph node examination, regional lymph nodes pathologically positive or regional lymph node surgery. In Table 3, Table A3 further information on the multivariate analyses can be found.

### 3.7 Therapy

Univariate analysis of therapeutic procedures is also shown in Table 1. In the analysis, factors correlating with OS and DSS were time from diagnosis to treatment, chemotherapy, radiotherapy, and surgery of primary site. Performance of therapeutic interventions correlated with better rates of survival. In the multivariate analysis that included therapy data, variables independently associated with OS and DSS were size, and the therapeutic interventions. More details can be found in Table A3.

## 4 Discussion

In the present study, data of 990 patients of all ages with RMS in the site OTH from the SEER 17 database was analyzed, with the primary objective to assess whether it is appropriate to treat OTH as a uniform site. Given the heterogeneity and the cross‐group categorization as unfavorable, the site was divided into granular subsites to then define their prognostic significance. Our results showed that there are major significant differences in the prognosis among the various subsites. The origin of the tumor within the OTH localization is an important independent prognostic factor. We were able to identify an OTH favorable subgroup. The OTH favorable group consists of the anal region, gallbladder, biliary tract, and breast, whereas the liver, lung and pleura and mediastinum have a remarkably poor prognosis. Thus, we question whether the categorization of OTH as a singular group is justifiable or if adjustments to the stratification system should be made. Moreover, within the OTH group, pathological examination of regional lymph nodes is independently associated with an improved prognosis.

Historically, RMS were classified into four types, microscopically: botryoid, alveolar, embryonal and pleomorphic [20]. Additionally, they were classified into clinical disease groups, which solely relied on the assessment of disease extent and resectability [13, 21, 22]. However, the need for a uniform staging classification system, which includes prognostic factors, quickly became evident [8, 10, 23]. The effects of tumor site on survival have long been known [8, 9, 13, 14]. The different localizations were defined and risk‐stratified into favorable and unfavorable. Sites considered favorable include the orbit, head and neck (excluding parameningeal), and genitourinary system (excluding prostate and bladder). All other sites are deemed unfavorable, with the evaluation of the biliary tract and liver evolving over time [10, 11, 12]. The site OTH is a heterogenous site comprising tumors arising from paraspinal or perianal regions or originating from the thorax (e.g., pleura, mediastinum, lung, breast, and diaphragm), abdomen (e.g., liver, biliary duct, pancreas, intestine, and retroperitoneum), pelvis or perineum. Since the development of a common classification system, the site OTH has consistently been deemed unfavorable [9, 10, 13, 14]. Only recently the biliary tract was seen as an exception within this site group, with the current FaR‐RMS Protocol downgrading the biliary tumors to a favorable site [EudraCT: 2018‐000515‐24].

The diverse origins of tumors within this group demonstrate the importance of determining the prognostic factor of the primary sites within the OTH group and raise questions as to whether this site should be divided into different subgroups, similar to the head/neck localization [24].

To the best of our knowledge, it is currently not possible, within existing literature, to compare the distribution of factors within the cohort, as no specific literature on the OTH localization in RMS exist. Additionally, only few studies on the occurrence of RMS in adults are available. For this reason, the literature on RMS in all sites, and especially in children, is here used as a reference.

A slight male predominance (56.9%) in this cohort, in accordance with most literature, was shown [4, 6, 15, 25, 26, 27]. The cohort consisted to a large extent of children and adolescents (54.2%) and had a peak incidence in the age group 0–9 years (34.7%). These findings reflect the observation that RMS is primarily a tumor type found in young individuals [6, 15, 23, 25, 26, 27] and is in alignment with known data comparing adult and pediatric cases [4].

The identification of the largest histological subgroups in this assessment, embryonal (33.0%) and alveolar (25.5%) RMS, is consistent with preexisting literature on the subject [4, 15, 26, 27, 28]. Existing literature does not provide sufficient evidence to make a clear statement regarding the similitude of size distribution of OTH RMS, as few uniform results on size exist. Tumors at the OTH site are assumably larger in size due to late clinical symptoms. However, there seems to be the indication, that RMS regardless of site are mainly > 5 cm, which was also shown in this analysis [15, 25, 27]. Given the lack of studies on the origins of RMS within the OTH localization, the most common site of occurrence within the OTH localization is hardly described.

In this cohort, most tumors originated in the pelvic area (site classification 1: 41.5%). For the site OTH, 5%–10% lymph node involvement was reported [23, 29, 30]. Overall, 23% of patients underwent pathological examination of regional lymph nodes, with 9.4% positive regional lymph nodes detected in pathology. Therefore, this localization is significantly below the normal values for lymph node involvement in RMS [6, 15, 23, 25, 29, 30]. In contrast to the stage distribution of RMS described in the literature, the OTH cohort exhibits a higher prevalence of the distant stage (43.8%) [4, 27].

Whereas prognostic factors within different RMS sites were investigated intensively [24, 31, 32, 33, 34, 35, 36], the OTH site was rarely analyzed, despite the known poor outcome.

In the pediatric groups (0–9 and 10–17 years), most tumors were 5–10 cm in size, with a slight predominance in the distant stage. Embryonal RMS, NOS and alveolar RMS were the most common histological types in these pediatric age groups. The most common site was the pelvic area. However, the abdomen and retroperitoneum (site classification 1) was a notable site for children (0–9 years) as well. Regional lymph nodes were examined pathologically more frequently in the pediatric groups compared to adults, although in many cases, they were either not examined or the examination status remained unknown. Furthermore, regional lymph nodes were mostly pathologically negative in children (0–9 years), but slightly more often positive in the adolescence (10–17 years).

In the adult group (≥ 18 years), most tumors were > 10 cm in size and distant in stage. The predominant histological type in this group, besides RMS, NOS, was pleomorphic RMS, adult type. In the adult cohort, the most common site was the pelvic area. Regional lymph nodes were often not examined, indicating no clear majority in terms of pathologically positive or negative regional lymph nodes.

In the age‐spanning multivariate analysis, independent prognostic factors were age, histological type, OTH subsite, stage, examination of regional lymph nodes, and pathologically positive regional lymph nodes.

Age is a well‐known independent factor in RMS [1, 6, 7, 8, 9, 25, 26, 37], yet evaluations across different age groups remain challenging due to the distinct trial inclusion criteria and the different treatment approaches in pediatric and adult oncology. In this analysis, embryonal RMS had the best outcome, while alveolar RMS showed the poorest outcomes. These findings are consistent with existing literature [6, 7, 25, 27, 28, 37, 38, 39]. Comparably little is known about the pleomorphic adult type, which in this study showed a slightly better outcome than the alveolar RMS. The histology as an independent prognostic factor was of particular interest in this study, since, for instance, pleomorphic RMS, adult type is commonly not included in pediatric clinical trials. Size was not an independent prognostic factor in the multivariate analysis, contrary to what was reported in the literature regarding RMS generally. This contradiction may arise from the lack of studies focusing on the impact of size at the OTH site. Notably, cases with unknown size (*n* = 316) were included as a separate group in the analyses and could contribute to these findings as well. In alignment with existing literature, worse outcomes were associated with larger size [28, 39, 40, 41]. The stage of disease, as is widely known, was an independent prognostic factor, where more advanced stages correlate with worse outcomes [25, 28, 37, 39, 40]. Although lymph node involvement is already recognized as an important prognostic factor with RMS [23, 28, 29, 39, 42], the role of pathological workup has received little attention thus far. This study demonstrated the statistical significance of both factors.

A limitation of our site evaluation is a certain subgroup, in which the precise origin within OTH cannot be further specified, namely C49.4‐Conn, subcutaneous, other soft tis: abdomen, C49.5‐Conn, subcutaneous, other soft tis: pelvis, C49.3‐Conn, subcutaneous, other soft tis: thorax, and C49.6‐Conn, subcutaneous, other soft tis: trunk, NOS. Therefore, they were included as separate groups to the analyses. A granular site classification inevitably requires the formation of small subgroups in the variable “site classification” and may therefore limit the informative value of the results and restricts the ability to provide definitive prognostic conclusions. Further research is needed to support the results, as well as to better understand the prognostic variability within the OTH localization to improve treatment strategies. With regard to lymph nodes in the OTH site, the lymphatic drainage routes and the involvement of lymph nodes can be uncertain, as identifying particular sentinel lymph nodes is often not possible. Another limitation was the retrospective design within an epidemiological registry. However, this type of study design made an age‐spanning analysis possible with long term follow‐up.

We can conclude that the OTH site should not be uniformly considered unfavorable, as our study shows notable prognostic differences. The OTH favorable group identified in this study includes the anal region, gallbladder, biliary tract, and breast. In contrast, the liver, lung and pleura and mediastinum showed a remarkably poor prognosis. These findings suggest the necessity of a change in risk stratification for RMS of the OTH site to avoid potential overtreatment of patients. Given the significant impact of regional lymph node examination and pathologically proven involvement of regional lymph nodes on prognosis, the retrieval of regional lymph nodes, with subsequent pathological assessment, is recommended for the OTH site.

## Author Contributions

**Leonie Kern:** conceptualization (supporting), data curation (lead), formal analysis (lead), investigation (equal), methodology (equal), resources (supporting), software (lead), validation (equal), visualization (lead), writing – original draft (lead), writing – review and editing (supporting). **Anton Henssen:** resources (supporting), validation (supporting), writing – review and editing (supporting). **Angelika Eggert:** resources (supporting), supervision (supporting), validation (supporting), writing – review and editing (supporting). **Monika Scheer:** conceptualization (lead), data curation (equal), formal analysis (supporting), investigation (equal), methodology (equal), project administration (lead), resources (equal), supervision (lead), validation (supporting), visualization (supporting), writing – original draft (supporting), writing – review and editing (lead).

## Conflicts of Interest

The authors declare no conflicts of interest.

## Supporting information


Figure A1.

## Data Availability

The data that support the findings of this study are openly available at https://seer.cancer.gov/.

## Appendix A1 A

**TABLE A1:** List of the causes of death.

Dead (attributable to this cancer dx) 92.4% (548)	% (*n*)
Soft tissue	40.9% (405)
Lung and bronchus	2.7% (27)
Miscellaneous neoplasms	2.6% (26)
Miscellaneous hematopoietic neoplasms	1.4% (14)
Retroperitoneum and peritoneum	1.1% (11)
Bones and joints	0.8% (8)
Corpus uteri	0.6% (6)
Heart, mediastinum, and pleura	0.6% (6)
Stomach	0.6% (6)
Liver	0.5% (5)
Gum	0.3% (3)
Prostate	0.3% (3)
Urinary bladder	0.3% (3)
Brain (malignant)	0.2% (2)
Breast	0.2% (2)
Kidney parenchyma	0.2% (2)
Ovary	0.2% (2)
Pancreas	0.2% (2)
Testis	0.2% (2)
Adrenal gland	0.1% (1)
Brain, CNS other, and intracranial gland (benign and borderline)	0.1% (1)
Cervix	0.1% (1)
Endocrine other	0.1% (1)
Esophagus	0.1% (1)
Extrahepatic bile ducts	0.1% (1)
Other digestive organs	0.1% (1)
Other urinary organs	0.1% (1)
Thyroid	0.1% (1)
Vulva	0.1% (1)

Dead of other causes 7.6% (45)	% (*n*)
Other cause of death	22.9% (11)
Dead (missing/unknown COD)	12.5% (6)
Ischemic heart disease	12.5% (6)
Diseases of arteries, arterioles and capillaries	6.3% (3)
Accidents and adverse effects	4.2% (2)
Diabetes mellitus	4.2% (2)
Soft tissue	4.2% (2)
Alzheimers (ICD‐9 and 10 only)	2.1% (1)
Bones and joints	2.1% (1)
Chronic liver disease and cirrhosis	2.1% (1)
Hodgkin lymphoma	2.1% (1)
Kaposi sarcoma	2.1% (1)
Large B‐cell lymphoma	2.1% (1)
Liver	2.1% (1)
Melanoma of the skin	2.1% (1)
Nephritis, nephrotic syndrome and nephrosis	2.1% (1)
Other and unspecified disorders of the circulatory system	2.1% (1)
Other infectious and parasitic diseases including HIV	2.1% (1)
Other leukemias	2.1% (1)
Pancreas	2.1% (1)
Precursor lymphoid neoplasms	2.1% (1)
Septicemia	2.1% (1)
Stomach	2.1% (1)

*Note:* Dead *n* = 593 (59.9%); of those: Dead (attributable to this cancer dx) *n* = 548 (92.4%), Dead (attributable to causes other than this cancer dx) + Dead (missing/unknown COD) *n* = 45 (7.6%).

**TABLE A2:** Site classifications and subgroups.

Site classification	Site grouping	Primary site labeled
1	Abdomen and retroperitoneum	C16.3‐Gastric antrum C16.8‐Overlapping lesion of stomach C16.9‐Stomach, NOS C17.2‐Ileum C17.9‐Small intestine, NOS C22.0‐Liver C23.9‐Gallbladder C24.0‐Extrahepatic bile duct C24.9‐Biliary tract, NOS C25.0‐Head of pancreas C25.1‐Body of pancreas C48.0‐Retroperitoneum C48.1‐Specified parts of peritoneum C48.2‐Peritoneum, NOS C48.8‐Overlapping lesion of retroperitoneum and peritoneum C49.4‐Conn, subcutaneous, other soft tis: abdomen C64.9‐Kidney, NOS C67.7‐Urachus C74.0‐Cortex of adrenal gland
	Thorax	C15.0‐Cervical esophagus C15.5‐Lower third of esophagus C34.0‐Main bronchus C34.1‐Upper lobe, lung C34.3‐Lower lobe, lung C34.8‐Overlapping lesion of lung C34.9‐Lung, NOS C38.0‐Heart C38.1‐Anterior mediastinum C38.2‐Posterior mediastinum C38.3‐Mediastinum, NOS C38.4‐Pleura, NOS C38.8‐Overlapping lesion of heart, mediastinum and pleura C49.3‐Conn, subcutaneous, other soft tis: thorax C50.1‐Central portion of breast C50.4‐Upper‐outer quadrant of breast C50.5‐Lower‐outer quadrant of breast C50.8‐Overlapping lesion of breast C50.9‐Breast, NOS
	Pelvis	C21.0‐Anus, NOS C21.8‐Overlapping lesion of rectum, anus, and anal canal C41.4‐Pelvic bones, sacrum, coccyx and associated joints C47.5‐Periph nerves and autonomic nervous system: pelvis C49.5‐Conn, subcutaneous, other soft tis: pelvis C65.9‐Renal pelvis
	Dorsum	C41.2‐Vertebral column C72.0‐Spinal cord
	Other	C44.5‐Skin of trunk C49.6‐Conn, subcutaneous, other soft tis: trunk, NOS C72.9‐Nervous system, NOS
2	Breast	C50.1‐Central portion of breast C50.4‐Upper‐outer quadrant of breast C50.5‐Lower‐outer quadrant of breast C50.8‐Overlapping lesion of breast C50.9‐Breast, NOS
	GI‐Tract (incl. rectum and anus)	C15.0‐Cervical esophagus C15.5‐Lower third of esophagus C16.3‐Gastric antrum C16.8‐Overlapping lesion of stomach C16.9‐Stomach, NOS C17.2‐Ileum C17.9‐Small intestine, NOS C21.0‐Anus, NOS C21.8‐Overlapping lesion of rectum, anus, and anal canal
	Kidney and adrenal gland	C64.9‐Kidney, NOS C65.9‐Renal pelvis C74.0‐Cortex of adrenal gland
	Liver and biliary	C22.0‐Liver C23.9‐Gallbladder C24.0‐Extrahepatic bile duct C24.9‐Biliary tract, NOS
	Lung	C34.0‐Main bronchus C34.1‐Upper lobe, lung C34.3‐Lower lobe, lung C34.8‐Overlapping lesion of lung C34.9‐Lung, NOS
	Mediastinum and pleura	C38.0‐Heart C38.1‐Anterior mediastinum C38.2‐Posterior mediastinum C38.3‐Mediastinum, NOS C38.4‐Pleura, NOS C38.8‐Overlapping lesion of heart, mediastinum and pleura
	Pancreas	C25.0‐Head of pancreas C25.1‐Body of pancreas
	Pelvis other	C41.4‐Pelvic bones, sacrum, coccyx and associated joints C47.5‐Periph nerves and autonomic nervous system: pelvis
	Peritoneum and retroperitoneum	C48.0‐Retroperitoneum C48.1‐Specified parts of peritoneum C48.2‐Peritoneum, NOS C48.8‐Overlapping lesion of retroperitoneum and peritoneum
	Skin of trunk	C44.5‐Skin of trunk
	Spinal	C41.2‐Vertebral column C72.0‐Spinal cord C72.9‐Nervous system, NOS
	Tissue abdomen (incl. urachus)	C49.4‐Conn, subcutaneous, other soft tis: abdomen C67.7‐Urachus
	Tissue pelvis	C49.5‐Conn, subcutaneous, other soft tis: pelvis
	Tissue thorax	C49.3‐Conn, subcutaneous, other soft tis: thorax
	Tissue trunk	C49.6‐Conn, subcutaneous, other soft tis: trunk, NOS
3	Parenchymatous organs	C22.0‐Liver C25.0‐Head of pancreas C25.1‐Body of pancreas C34.1‐Upper lobe, lung C34.3‐Lower lobe, lung C34.8‐Overlapping lesion of lung C34.9‐Lung, NOS C64.9‐Kidney, NOS C74.0‐Cortex of adrenal gland
	Hollow organs	C15.0‐Cervical esophagus C15.5‐Lower third of esophagus C16.3‐Gastric antrum C16.8‐Overlapping lesion of stomach C16.9‐Stomach, NOS C17.2‐Ileum C17.9‐Small intestine, NOS C23.9‐Gallbladder C24.0‐Extrahepatic bile duct C24.9‐Biliary tract, NOS C34.0‐Main bronchus C38.0‐Heart C67.7‐Urachus
	Cavities	C38.1‐Anterior mediastinum C38.2‐Posterior mediastinum C38.3‐Mediastinum, NOS C38.4‐Pleura, NOS C38.8‐Overlapping lesion of heart, mediastinum and pleura C48.0‐Retroperitoneum C48.1‐Specified parts of peritoneum C48.2‐Peritoneum, NOS C48.8‐Overlapping lesion of retroperitoneum and peritoneum C65.9‐Renal pelvis
	Tissue abdomen	C49.4‐Conn, subcutaneous, other soft tis: abdomen
	Tissue pelvis	C49.5‐Conn, subcutaneous, other soft tis: pelvis
	Tissue thorax	C49.3‐Conn, subcutaneous, other soft tis: thorax
	Tissue trunk	C44.5‐Skin of trunk C49.6‐Conn, subcutaneous, other soft tis: trunk, NOS C50.1‐Central portion of breast C50.4‐Upper‐outer quadrant of breast C50.5‐Lower‐outer quadrant of breast C50.8‐Overlapping lesion of breast C50.9‐Breast, NOS
	Other	C21.0‐Anus, NOS C21.8‐Overlapping lesion of rectum, anus, and anal canal C41.2‐Vertebral column C41.4‐Pelvic bones, sacrum, coccyx and associated joints C47.5‐Periph nerves and autonomic nervous system: pelvis C72.0‐Spinal cord C72.9‐Nervous system, NOS
4	OTH favorable	C21.0‐Anus, NOS C21.8‐Overlapping lesion of rectum, anus, and anal canal C23.9‐Gallbladder C24.0‐Extrahepatic bile duct C24.9‐Biliary tract, NOS C50.1‐Central portion of breast C50.4‐Upper‐outer quadrant of breast C50.5‐Lower‐outer quadrant of breast C50.8‐Overlapping lesion of breast C50.9‐Breast, NOS
	Heart	C38.0‐Heart
	Intestines	C15.5‐Lower third of esophagus C16.3‐Gastric antrum C16.8‐Overlapping lesion of stomach C16.9‐Stomach, NOS C17.2‐Ileum C17.9‐Small intestine, NOS
	Kidney	C64.9‐Kidney, NOS C65.9‐Renal pelvis
	Liver	C22.0‐Liver
	Lung and pleura	C34.0‐Main bronchus C34.1‐Upper lobe, lung C34.3‐Lower lobe, lung C34.8‐Overlapping lesion of lung C34.9‐Lung, NOS C38.4‐Pleura, NOS
	Mediastinum	C15.0‐Cervical esophagus C38.1‐Anterior mediastinum C38.2‐Posterior mediastinum C38.3‐Mediastinum, NOS C38.8‐Overlapping lesion of heart, mediastinum and pleura
	Pancreas	C25.0‐Head of pancreas C25.1‐Body of pancreas
	Pelvis and vertebral	C41.2‐Vertebral column C41.4‐Pelvic bones, sacrum, coccyx and associated joints C72.0‐Spinal cord
	Peritoneum	C48.1‐Specified parts of peritoneum C48.2‐Peritoneum, NOS
	Retroperitoneum	C48.0‐Retroperitoneum C48.8‐Overlapping lesion of retroperitoneum and peritoneum C74.0‐Cortex of adrenal gland
	Tissue abdomen	C49.4‐Conn, subcutaneous, other soft tis: abdomen C67.7‐Urachus
	Tissue pelvis	C47.5‐Periph nerves and autonomic nervous system: pelvis C49.5‐Conn, subcutaneous, other soft tis: pelvis
	Tissue thorax	C49.3‐Conn, subcutaneous, other soft tis: thorax
	Tissue trunk	C44.5‐Skin of trunk C49.6‐Conn, subcutaneous, other soft tis: trunk, NOS C72.9‐Nervous system, NOS

**TABLE A3:** Multivariate analysis of patients', tumor, and therapy characteristics.

	Overall survival				Disease specific survival			
	Hazard ratio	95% CI, lower	95% CI, upper	*p*	Hazard ratio	95% CI, lower	95% CI, upper	*p*
Age, years								
0–9	1.0			**< 0.001**	1.0			**< 0.001**
10–17	1.197	0.910	1.574	0.198	1.242	0.937	1.646	0.131
18–39	1.442	1.082	1.921	**0.012**	1.521	1.132	2.042	**0.005**
40–64	2.834	2.114	3.799	**< 0.001**	2.927	2.158	3.970	**< 0.001**
≥ 65	4.582	3.323	6.319	**< 0.001**	4.248	3.025	5.967	**< 0.001**
Histological type								
Embryonal RMS, NOS	1.0			**< 0.001**	1.0			**< 0.001**
Mixed type RMS	1.581	0.731	3.419	0.245	1.755	0.809	3.809	0.155
Spindle cell RMS	1.580	1.020	2.448	**0.040**	1.574	0.991	2.499	0.055
Alveolar RMS	1.700	1.309	2.206	**< 0.001**	1.741	1.329	2.281	**< 0.001**
Pleomorphic adult type	1.784	1.287	2.471	**< 0.001**	1.784	1.266	2.513	**< 0.001**
RMS, NOS	1.787	1.363	2.343	**< 0.001**	1.769	1.331	2.351	**< 0.001**
Stage								
Localized	1.0			**< 0.001**	1.0			**< 0.001**
Regional	1.489	1.121	1.978	**0.006**	1.653	1.219	2.242	**0.001**
Distant	2.967	2.262	3.893	**< 0.001**	3.321	2.479	4.451	**< 0.001**
Unstaged	1.159	0.743	1.809	0.514	1.093	0.667	1.793	0.724
Size, cm								
< 5	1.0			**0.017**	1.0			**0.012**
5–10	1.086	0.762	1.547	0.649	0.966	0.668	1.398	0.856
> 10	1.481	1.040	2.109	**0.030**	1.384	0.958	1.998	0.083
Unknown/Not reported	1.356	0.956	1.924	0.088	1.278	0.890	1.837	0.184
Surgery primary site								
Yes	1.0			**< 0.001**	1.0			**< 0.001**
No	2.126	1.749	2.584	**< 0.001**	2.104	1.716	2.579	**< 0.001**
Unknown	1.279	0.307	5.321	0.735	1.612	0.385	6.753	0.514
Radiation								
Yes	1.0			**< 0.001**	1.0			**0.003**
No	1.434	1.196	1.720	**< 0.001**	1.388	1.150	1.676	**< 0.001**
Recommended, unknown if administered	0.979	0.496	1.932	0.951	0.988	0.500	1.952	0.973
Chemotherapy								
Yes	1.0				1.0			
No/Unknown	1.727	1.371	2.176	**< 0.001**	1.677	1.311	2.146	**< 0.001**
Age, years								
0–9	1.0			**< 0.001**	1.0			**< 0.001**
10–17	1.213	0.922	1.596	0.168	1.258	0.949	1.668	0.110
18–39	1.490	1.117	1.989	**0.007**	1.575	1.171	2.118	**0.003**
40–64	2.819	2.102	3.780	**< 0.001**	2.907	2.143	3.945	**< 0.001**
≥ 65	4.540	2.288	6.268	**< 0.001**	4.193	2.982	5.898	**< 0.001**
Histological type								
Embryonal RMS, NOS	1.0			**< 0.001**	1.0			**< 0.001**
Mixed type RMS	1.565	0.723	3.386	0.255	1.733	0.798	3.761	0.165
Spindle cell RMS	1.592	1.028	2.467	**0.037**	1.585	0.999	2.517	0.051
Alveolar RMS	1.722	1.325	2.239	**< 0.001**	1.770	1.348	2.322	**< 0.001**
Pleomorphic adult type	1.798	1.298	2.491	**< 0.001**	1.798	1.277	2.532	**< 0.001**
RMS, NOS	1.830	1.394	2.403	**< 0.001**	1.815	1.365	2.415	**< 0.001**
Stage								
Localized	1.0			**< 0.001**	1.0			**< 0.001**
Regional	1.498	1.125	1.995	**0.006**	1.668	1.227	2.267	**0.001**
Distant	2.973	2.267	3.898	**< 0.001**	3.330	2.486	4.461	**< 0.001**
Unstaged	1.192	0.763	1.862	0.441	1.129	0.687	1.855	0.631
Size, cm								
< 5	1.0			**0.020**	1.0			**0.014**
5–10	1.084	0.761	1.543	0.656	0.967	0.669	1.399	0.861
> 10	1.462	1.026	2.083	**0.035**	1.366	0.946	1.973	0.096
Unknown/Not reported	1.368	0.964	1.941	0.080	1.292	0.898	1.858	0.167
Surgery primary site								
Yes	1.0			**< 0.001**	1.0			**< 0.001**
No	2.066	1.689	2.525	**< 0.001**	2.032	1.647	2.506	**< 0.001**
Unknown	1.718	0.390	7.563	0.474	2.288	0.511	10.246	0.279
Radiation								
Yes	1.0			**< 0.001**	1.0			**0.003**
No	1.423	1.187	1.706	**< 0.001**	1.376	1.140	1.661	**< 0.001**
Recommended, unknown if administered	0.945	0.478	1.867	0.871	0.948	0.479	1.876	0.878
Chemotherapy								
Yes	1.0				1.0			
No/Unknown	1.741	1.378	2.199	**< 0.001**	1.697	1.323	2.178	**< 0.001**
Regional lymph node surgery								
Yes	1.0			0.258	1.0			0.186
No	1.093	0.837	1.429	0.513	1.122	0.850	1.479	0.417
Unknown/Not reported	0.750	0.439	1.280	0.292	0.718	0.404	1.275	0.259

*Note:* Bold values indicate statistical significance *p* < 0.05.
