# Supplementary figures and images for: The Other Site of Rhabdomyosarcoma
Source: Cancer Med. 2024 Oct 28;13(20):e70348. doi: 10.1002/cam4.70348 (PMC11513437; doi:10.1002/cam4.70348)

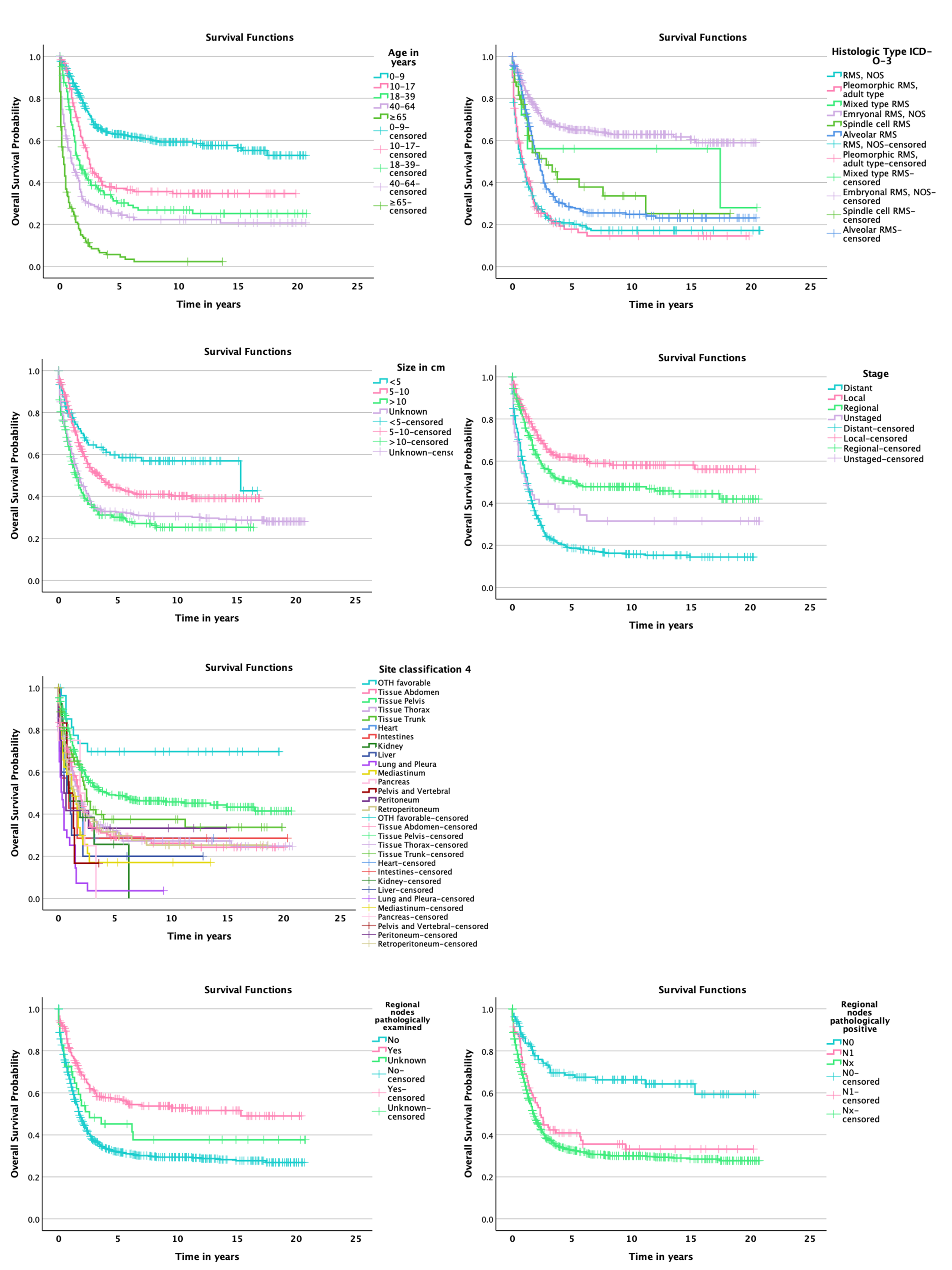

Supplement: Supplementary file 1 — Figure A1. [file CAM4-13-e70348-s001.tif]
